# Dysregulated Coagulation and Fibrinolysis Are Present in Patients Admitted to the Emergency Department with Acute Hypoxemic Respiratory Failure: A Prospective Study

**DOI:** 10.3390/biomedicines12051081

**Published:** 2024-05-13

**Authors:** Chrysi Keskinidou, Alice Georgia Vassiliou, Elena Papoutsi, Edison Jahaj, Ioanna Dimopoulou, Ilias Siempos, Anastasia Kotanidou

**Affiliations:** First Department of Critical Care Medicine & Pulmonary Services, School of Medicine, National and Kapodistrian University of Athens, Evangelismos Hospital, 106 76 Athens, Greece; chrysakes29@gmail.com (C.K.); helenapapoutsi@gmail.com (E.P.); edison.jahaj@gmail.com (E.J.); idimo@otenet.gr (I.D.); isiempos@yahoo.gr (I.S.)

**Keywords:** AHRF, plasminogen, sEPCR, coagulation, fibrinolysis, endothelium

## Abstract

Acute hypoxemic respiratory failure (AHRF) is defined as acute and progressive, and patients are at a greater risk of developing acute respiratory distress syndrome (ARDS). Until now, most studies have focused on prognostic and diagnostic biomarkers in ARDS. Since there is evidence supporting a connection between dysregulated coagulant and fibrinolytic pathways in ARDS progression, it is plausible that this dysregulation also exists in AHRF. The aim of this study was to explore whether levels of soluble endothelial protein C receptor (sEPCR) and plasminogen differentiate patients admitted to the emergency department (ED) with AHRF. sEPCR and plasminogen levels were measured in 130 AHRF patients upon ED presentation by ELISA. Our results demonstrated that patients presenting to the ED with AHRF had elevated levels of sEPCR and plasminogen. It seems that dysregulation of coagulation and fibrinolysis occur in the early stages of respiratory failure requiring hospitalisation. Further research is needed to fully comprehend the contribution of sEPCR and plasminogen in AHRF.

## 1. Introduction

Respiratory failure (RF) is characterised by the inability of the lungs to either adequately provide oxygen to the body, causing hypoxemia, or sufficiently remove carbon dioxide from the body, leading to hypercapnia [1]. Acute respiratory failure (ARF) can be caused by respiratory, cardiovascular, or systemic disease, and is characterised by acute and increasing hypoxemia that occurs in previously healthy individuals. ARF is a heterogeneous syndrome, associated with high hospital morbidity and mortality rates [2]. In the absence of chronic hypoxemic respiratory failure (requiring long-term oxygen therapy at home), patients diagnosed with de novo hypoxemic respiratory failure suffer from significant hypoxemia and tachypnoea [3].

Acute hypoxemic respiratory failure (AHRF) is defined as acute and progressive. Hence, patients are at a greater risk of developing acute respiratory distress syndrome (ARDS) [4]. Using the Berlin definition, ARDS is diagnosed by early onset or worsening of the respiratory symptoms, the presence of bilateral opacities on chest imaging, and non-cardiogenic respiratory failure, leading to mild, moderate, or severe oxygen impairment (PaO_2_/FIO_2_ ≤ 300 mmHg) [5]. Pathophysiologically, it is characterised by damage to the capillary endothelium and alveolar epithelium, and fluid accumulation in the alveolar space, leading to alveolar oedema. The European Society of Intensive Care Medicine (ESICM) clinical practice guideline (CPG) recommends that patients with AHRF should receive high-flow nasal cannula (HFNC) oxygen and not conventional oxygen therapy to reduce the risk of intubation [6]. Recently, a new global definition of ARDS, based on the Berlin definition, was proposed, to include patients receiving non-invasive support [7]. In this revised definition, the authors suggested a new ARDS category consisting of non-intubated ARDS patients who are on high-flow nasal oxygenation (HFNO) or non-invasive ventilation at the time of diagnosis, with an oxygen delivery threshold of 30 L/min with HFNO [7].

Over the past years, several endothelial biomarkers have been assessed as possible therapeutic targets, as well as in diagnosing and monitoring ARDS patients; however, little progress has been made. Considering the underlying molecular pathophysiological mechanisms, finding generalisable biomarkers could lead to the development of a gold-standard diagnostic biomarker panel or could help patients’ clinical stratification [8]. In patients with ARDS, both the coagulation cascade and the fibrinolytic system are activated systemically and, in the lung, favour the formation of fibrin clots [9]. Since there is evidence supporting a connection between dysregulated coagulant and fibrinolytic pathways in ARDS progression [10,11], it is plausible that this dysregulation also exists in AHRF. Most studies have examined the disruption of the abovementioned pathways in established ARDS; hence, the aim of the current study was to explore whether there is a connection between AHRF and two key components of coagulation and fibrinolysis.

The protein C pathway is known for its anticoagulant and cytoprotective activities. To maintain vascular haemostasis and regulate the inflammatory response, protein C needs to be activated. Membrane-bound endothelial cell protein C receptor (EPCR) is the key receptor of protein C activation by the thrombin–thrombomodulin (TM) complex [12]. However, in the presence of inflammatory mediators, EPCR is cleaved in its soluble (s) form in the circulation. sEPCR inhibits the anticoagulant activity of the activated protein C (APC) [13]. On the other hand, plasminogen (PLG) is the zymogen form of plasmin and has a central role in the fibrinolytic pathway. The conversion of PLG to plasmin, the primary fibrinolysin, is mediated via the action of two plasminogen activators, tissue-type plasminogen activator (tPA) and urokinase-type plasminogen activator (uPA) [14]. Data from clinical and in vivo studies indicate that PLG also contributes to the regulation of the inflammatory response [15].

In previous studies, we have demonstrated the promising prognostic capability of sEPCR levels in sepsis and COVID-19 [16,17,18], while we also found that critically ill COVID-19 non-survivors had higher PLG levels compared to survivors on ICU admission [19]. In view of the above, the purpose of the present study was to explore whether sEPCR and PLG levels differentiate patients presenting to the emergency department (ED) with AHRF, regardless of severe acute respiratory syndrome coronavirus 2 (SARS-CoV-2) infection, and to examine whether sEPCR and PLG levels could provide important clinical information regarding AHRF progression.

## 2. Materials and Methods

This observational single-centre study took place in the ED of “Evangelismos” Hospital from December 2021 to March 2023. The Research Ethics Committee of “Evangelismos” Hospital approved this study (476/7-10-2021) and all procedures were conducted in compliance with the Helsinki Declaration. Prior to study enrolment, informed consent was obtained from all subjects involved in this study.

The inclusion criteria were adults (>18 years) and the presence of de novo AHRF. De novo AHRF was considered if a patient required an oxygen flow rate of 5 L/min or more to maintain oxygen saturation (SpO_2_) levels > 90% in the absence of prior chronic RF. Exclusion criteria were no requirement for hospital admission, post-operative ARF (within one week), chronic hypoxemic RF (requiring long-term oxygen therapy at home), hypercapnic RF, transfer from another hospital or facility, pregnancy, admission to the hospital purely to facilitate comfort care, and lack of informed consent. ARDS was assessed according to the Berlin and the newly proposed definition [5]. SARS-CoV-2 infection was confirmed by real-time reverse transcription PCR (RT-PCR) in nasopharyngeal swabs. The quick sequential organ failure assessment (qSOFA) (respiratory rate of 22/min or greater, altered mentation, or systolic blood pressure of 100 mm Hg or less) was calculated in the patients presenting to the ED [20]. The enrolled patients with AHRF required hospitalisation and were disposed either in the general ward or in the ICU.

Following study enrolment, patients’ demographics, current medication, underlying medical conditions, qSOFA score and vital signs, clinical variables including airway management (the type of oxygen delivery device and arterial blood gases), AHRF-related variables such as predisposing risk factors and the presence of infiltrates in radiographic imaging, laboratory findings, and outcomes were recorded.

Moreover, a total of 30 SARS-CoV-2-negative, age- and sex-matched patients who visited the ED for another medical reason and did not require hospitalisation were recruited in the study and were used as the control group.

Blood samples were obtained within 6 h from ED presentation. Blood was drawn in BD Vacutainer™ Heparin Plasma Tubes (Becton, Dickinson and Company, Franklin Lakes, NJ, USA), portioned and stored into aliquots at −80 °C until used.

Soluble plasminogen (Wuhan Fine Biotech Co. Ltd., Wuhan, China; intra-assay coefficient of variability (CV) < 8%; detection limit = 46.875 pg/mL; assay range, 78.125–5000 pg/mL) and sEPCR (R&D Systems, Inc., Minneapolis, MN, USA; CV = 5.8%; detection = 0.3 ng/mL; assay range, 0.3–20 ng/mL) were measured in the plasma samples by enzyme-linked immunosorbent assay (ELISA) according to the manufacturers’ instructions. The assays used two different polyclonal antibodies against the molecules as catching and tagging antibodies. The researcher who performed the measurements was blinded to the samples measured. Prior to assaying, the appropriate sampling dilution was determined. Samples were diluted 1:10,000 for the measurement of soluble PLG, while for sEPCR, the samples were pre-treated with HCl and neutralised with NaOH (as per protocol instructions) and were subsequently diluted 1:20 (final dilution 1:39). All samples were assayed in duplicates.

Data are presented as individual values (*n*, %), mean ± standard deviation (SD) for normally distributed variables, and median with interquartile range (IQR) for variables with skewed distribution, as appropriate. Student’s *t*-test or the non-parametric Mann–Whitney test for skewed data was used for two group comparisons, while the chi-square test was performed for associations between qualitative variables. More than two group comparison were performed with one-way ANOVA, followed by Dunn’s multiple comparison test. Receiver operating characteristic (ROC) curves were plotted using the presence of AHRF as the classification variable and the biomarker levels on ED presentation as prognostic variables. The optimal cut-off value for predicting AHRF was calculated as the point with the greatest combined sensitivity and specificity. The IBM SPSS statistical package, version 22.0 (IBM Software Group, Armonk, NY, USA), and GraphPad Prism, version 8.0 (GraphPad Software, San Diego, CA, USA), were used for data analysis. *p*-values < 0.05 were considered statistically significant.

## 3. Results

### 3.1. Study Population

**Figure 1:**
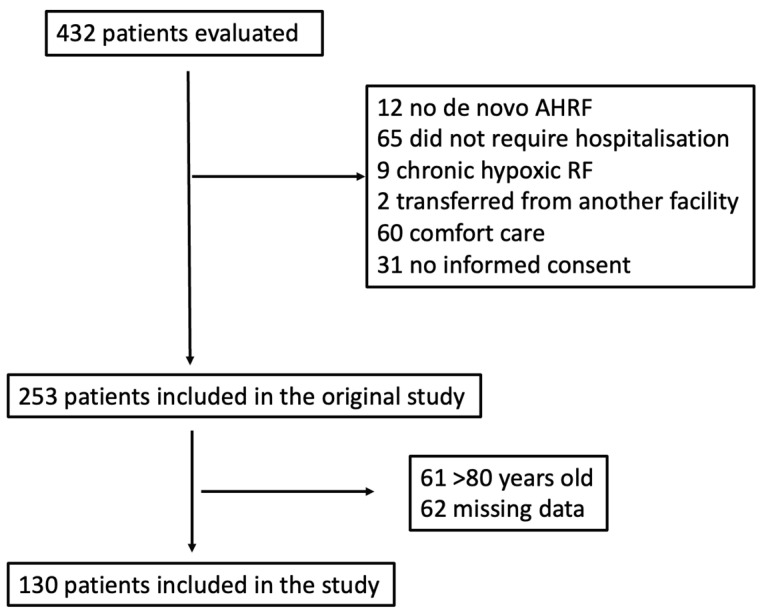
Study flow chart. AHRF = acute hypoxemic respiratory failure; RF = respiratory failure.

During the study period, 432 patients who presented to the ED were evaluated for inclusion in this study. According to the inclusion and exclusion criteria, 253 were included in the original study. Of these, 61 patients were over 80 years old, and we decided not to measure endothelial biomarkers in these patients, since endotheliopathy is pronounced in elderly frail patients. In 62 patients, we had missing data. Hence, 130 AHRF patients were finally included in the measurements. The study flow chart is shown in Figure 1.

All enrolled patients with AHRF required hospitalisation and were disposed either in the general ward (*n* = 103) or in the ICU (*n* = 27). The median age of our patients was 67 years, nearly half were male (48.5%), and 78.5% had comorbidities, with hypertension being the most prominent. Fifty-one patients were under medication for their underlying conditions. Most patients (56.9%) had a qSOFA score of zero. The major predisposing factor for AHRF was SARS-CoV-2 infection, with 73 patients testing positive (56%), followed by pulmonary infection (*n* = 36). One patient did not present with any AHRF predisposing factors. Table 1 presents the demographic and clinical characteristics of the study population. Thirty subjects (median age of 60 years, 57% male, and 83% with at least one comorbidity, with hypertension being the most prevalent) who presented to the ED without AHRF and did not require hospitalisation were also included for biomarker assessment.

### 3.2. AHRF and Biomarker Levels

**Figure 2:**
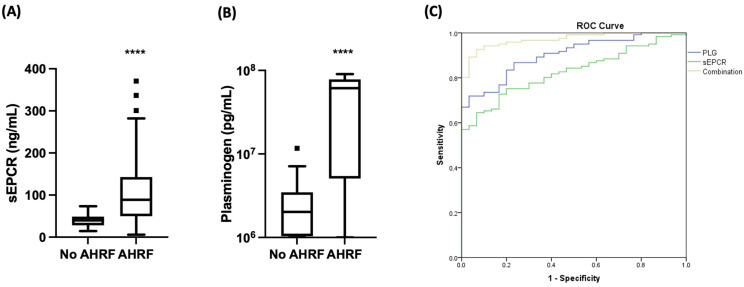
Emergency department (ED) presentation levels of sEPCR and PLG in patients with AHRF (*n* = 130) and the control group (*n* = 30). (**A**) sEPCR and (**B**) Plasminogen levels are presented as box plots. Line in the middle is the median value; lower and upper lines are the 25th and 75th centiles; whiskers are the range of values; bullets are outliers. Two-group comparisons were performed with the non-parametric Mann–Whitney test. **** *p* < 0.0001. (**C**) A receiver operating characteristic (ROC) curve analysis was generated using AHRF as the classifying variable. Blue line, PLG (sensitivity = 70%, specificity = 96.7%); green line, sEPCR (sensitivity = 64.5%, specificity = 93.3%); yellow line, combination of PLG and sEPCR (sensitivity = 89.3%, specificity = 96.7%). AHRF = acute hypoxemic respiratory failure; PLG = plasminogen; sEPCR = soluble endothelial protein C receptor.

Soluble (s) EPCR and PLG levels were concurrently measured in the plasma samples of the 130 AHRF patients and the control group. As seen in Figure 2, the levels of sEPCR were significantly elevated in the AHRF patients compared to the control group (88.92 ng/mL vs. 39.94 ng/mL, respectively, *p* < 0.0001, Figure 2A). Regarding PLG levels, similar results were observed; PLG levels were profoundly increased in the AHRF patients compared to the control group. More specifically, PLG levels were 61.29 × 10^6^ pg/mL vs. 2.02 × 10^6^ pg/mL, respectively (*p* < 0.0001, Figure 2B).

ROC curves were generated thereafter to test the prognostic accuracy of the biomarkers or their combination using AHRF as the classifying variable. sEPCR showed an area under the curve (AUC) of 0.83 (0.76–0.89) (*p* < 0.0001). A cut-off value of 66.60 ng/mL showed a sensitivity of 64.5% and a specificity of 93.3% for AHRF. Plasminogen levels showed an AUC of 0.90 (0.85–0.95) (*p* < 0.0001), with a cut-off value of 7.11 × 10^6^ pg/mL, showing a sensitivity of 70% and a specificity of 96.7%. The ROC curves had a similar prognostic accuracy (*p* > 0.05). The combination of the two biomarkers showed an AUC of 0.97 (0.95–0.99) (*p* < 0.0001). When we compared the ROC curve generated from the combination of the two biomarkers, we observed that it had a statistically significantly higher prognostic accuracy compared to either biomarker alone (sEPCR vs. combination, *p* < 0.0001, and PLG vs. combination, *p* = 0.002). Figure 2C depicts the ROC curves generated.

Since more than half of the AHRF patients had SARS-CoV-2 infection as a predisposing factor, we also analysed the levels of the two biomarkers only in the SARS-CoV-2-negative patients (*n* = 57) compared to the control group. Our results showed that the subset of the SARS-CoV-2-negative AHRF patients also had higher sEPCR and PLG levels compared to the control group (80.27 ng/mL vs. 39.94 ng/mL, *p* < 0.0001, and 59.97 × 10^6^ pg/mL vs. 2.02 × 10^6^ pg/mL, *p* < 0.0001, respectively). Of note, the AHRF SARS-CoV-2-positive patients tended to have higher sEPCR levels compared to the AHRF SARS-CoV-2-negative patients (105.80 ng/mL vs. 80.27 ng/mL, *p* = 0.1), while they had higher PLG levels (62.21 × 10^6^ pg/mL vs. 59.97 × 10^6^ pg/mL, *p* = 0.047). 

We subsequently compared the biomarker levels based on the patients’ disposition. ICU-admitted patients (*n* = 27) had similar sEPCR and PLG levels compared to general ward patients (*n* = 103), whereas both subgroups had higher levels compared to the control group (sEPCR, *p* < 0.01, and PLG, *p* < 0.0001).

Based on the Berlin definition, only intubated patients are included in the ARDS definition. The newly proposed global definition also includes non-intubated patients supported by HFNO. Taking these into consideration, we finally assigned the AHRF patients to two subgroups according to the subsequent development of ARDS, using both the Berlin definition (*n* = 29) and the newly proposed global definition (*n* = 45). We then compared the ED levels of sEPCR and soluble PLG within the AHRF subgroups and versus the control group. We found that, regardless of the definition used, AHRF patients who will develop ARDS and those who will not, had similar levels of both sEPCR and PLG on ED admission (*p* > 0.05); however, the AHRF patients who will subsequently either develop ARDS or not, had elevated ED levels compared to the control group (sEPCR, *p* < 0.01, and PLG, *p* < 0.0001).

## 4. Discussion

In the present study, we were able to show that patients admitted to the ED with AHRF present with elevated levels of sEPCR and plasminogen. It seems possible that dysregulation of the coagulation and fibrinolytic pathways may be present in the early stages of de novo AHRF requiring hospitalisation.

Patients who suffer from AHRF are at a greater risk of deteriorating to ARDS. Since 2012, the clinical diagnosis for ARDS has been based on the Berlin definition [5]. However, this definition does not include non-intubated ARDS patients. Recently, Matthay et al. suggested a new global definition of ARDS, revising the Berlin definition criteria to also include non-intubated patients supported by HFNO [7]. To date, most studies have examined the ability of various biomarkers to prognosticate or diagnose ARDS, post-ICU admission. Hence, in the present study, we aimed to measure selected coagulation and fibrinolysis biomarkers within 6 h from ED presentation in patients with AHRF to explore whether the disrupted coagulation and fibrinolysis pathways seen in ARDS are also present in AHRF. We also examined whether these biomarkers could possibly provide important clinical information regarding outcomes.

Over recent years, the number of biomarkers examined in critical illnesses, such as ARDS and sepsis, has continued to increase [16,21]. Based on previous studies of our group, we chose to measure sEPCR and PLG to observe early signs of abnormal coagulant and fibrinolytic function in patients presented to the ED.

The acute phase of ARDS is characterised by diffuse alveolar damage, including oedema and hyaline membrane formation and inflammatory lung injury [5]. Imbalance between the coagulation cascade and the inflammatory response plays a crucial role in ARDS pathogenesis [22]. The innate host response is further triggered by the damaged endothelial cells, leading to an interplay between the aggregated immune cells and platelets with the generated fibrin, a process also known as immunothrombosis, inducing the formation of microthrombi in the vasculature [23]. Moreover, the protein C pathway is a major component in regulating severe systemic inflammatory responses, including sepsis and ARDS [24]. However, little is known about the pathophysiology of AHRF.

In the presence of inflammatory signals, the haemostatic equilibrium shifts in favour of a prothrombotic and anti-fibrinolytic state [25]. Endothelial cells are considered the major regulators of vascular homeostasis. While, under normal circumstances, the endothelium is responsible for maintaining an anti-inflammatory, anti-thrombotic, and vasodilating phenotype, when endothelial dysfunction is established, endothelial cells trigger fibrin formation and enable platelet adhesion and aggregation [26].

Soluble endothelial protein C receptor haplotypes have been associated with higher plasma levels and higher thrombotic risk [27,28]. The exact role of sEPCR in ARDS has not been fully elucidated. Altered plasma levels of sEPCR have been associated with poor clinical outcomes in patients with ARDS. Previous studies demonstrated that in patients with the formerly used term acute lung injury (ALI) and in ARDS patients, protein C levels were disrupted and could be used as an independent predictor of mortality [29,30]. In lung specimens obtained from patients who died from severe malaria-associates ARDS, the anticoagulant properties, as characterised by EPCR and thrombomodulin, were dysregulated [31]. Moreover, the presence of specific genotypes in the *EPCR* gene have been associated with increased mortality in ARDS patients [32]. sEPCR has also been suggested as a key player in sepsis pathogenesis. In patients with severe pneumococcal pneumonia, high sEPCR levels on day 2 post-ICU admission were associated with poor sepsis outcomes [33]. In another study, serial measurements of plasma sEPCR levels indicated that a transient but significant increase in circulating sEPCR on day 2 of their ICU stay was associated with a poor 28-day outcome [34]. In a previous study, we demonstrated that ICU-admission sEPCR levels, in initially non-septic patients, could differentiate the patients who would eventually develop sepsis [16,18]. We recently suggested that sEPCR could be used as a point-of-care test in SARS-CoV-2-positive patients, as COVID-19 patients who required hospitalisation had higher sEPCR levels compared to COVID-19 outpatients [17]. In the present study, we observed that despite the presence or not of COVID-19, sEPCR levels were higher in the patients who presented to the ED with AHRF compared to the control group. Moreover, regardless of the definition used for ARDS diagnosis, sEPCR ED levels were comparable between the AHRF patients who will subsequently develop ARDS or not; however, both subgroups had higher sEPCR levels compared to the non AHRF control group. In addition, sEPCR levels did not differ according to the patients’ disposition (general ward or ICU). As opposed to the previous aforementioned studies that measured sEPCR in ARDS patients and found correlations with outcomes, we measured sEPCR on ED admission prior to the development of ARDS. Therefore, we suggest that sEPCR levels measured very early on ED admission, despite not providing us with information regarding poor outcomes, could represent an early index of endothelial dysfunction in AHRF.

Plasminogen, a glycoprotein located in the blood plasma, plays a role in ARDS through its involvement in the fibrinolytic system. Apart from its role in the resolution of blood clots, PLG also affects the resolution of the inflammatory response by regulating leukocyte recruitment and cytokine and chemokine production [35]. In previous studies, bronchoalveolar lavage (BAL) samples from adult patients with ARDS have shown high PLG and active plasmin levels, as well as high levels of the plasminogen activator inhibitor 1 (PAI-1) and lower levels of uPA [36]. In another study, the decreased fibrinolytic activity in BAL samples from ARDS patients was attributed to the inhibition of active plasmin and plasminogen activators, rather to the local insufficiency of PLG [37]. No differences in soluble PLG levels were found between critically ill COVID-19 patients and critically ill non-COVID-19 patients; however, the COVID-19 patients had higher PLG levels compared to healthy controls [38]. Similarly, circulating PLG levels were comparable in COVID-19 patients and healthy controls, as well as in non-ICU and ICU COVID-19 patients [39]. In another study, COVID-19 patients who required ICU admission had lower circulating PLG levels compared to COVID-19 patients who were discharged from the ED [40]. Finally, in a critically ill COVID-19 cohort, we showed that, on ICU admission, non-survivors had higher soluble PLG levels compared to survivors [19]. Herein, we found that patients presenting to the ED with AHRF had higher soluble PLG levels compared to the control group. Moreover, AHRF SARS-CoV-2-positive patients had higher PLG levels compared to the AHRF SARS-CoV-2-negative patients, but levels of both subgroups were higher than the control group. Furthermore, when the AHRF patients were divided based on the subsequent development of ARDS, using both the Berlin and the newly proposed definition, they showed comparable ED soluble PLG levels that were higher than the control group. Similarly to sEPCR, soluble PLG levels upon presentation to the ED did not differ according to the patients’ disposition. Our results are in line with previous studies showing no differences in soluble PLG levels in non-ICU and ICU patients. Studies until now have shown increased PLG in BAL samples from adult patients with ARDS. Our findings might imply that within 6 h from ED presentation, the disrupted fibrinolysis pathways usually seen in ARDS are also present in AHRF.

It is important to recognise our study’s limitations. Firstly, we only measured the soluble levels of EPCR and PLG in the circulation and did not include measurements from BAL samples to delineate whether the measured plasma concentrations are representative of the lung milieu. Secondly, our control group was rather small. Nevertheless, it consisted of patients whose characteristics were matched to the AHRF cohort. When sub-analyses were performed, the smaller subgroups also showed differences from the control group. Finally, we performed only one blood draw upon presentation to the ED; serial measurements would have been more useful in further exploring the prognostic ability of the selected biomarkers. However, as opposed to most studies investigating endothelial biomarkers in more severe RF, in the present study, the samples were obtained in the early stages of respiratory failure diagnosis (within 6 h post-ED presentation). Moreover, the patients had not received any treatment for their diagnosis. To the best of our knowledge, this is the first study to evaluate the complex interplay between inflammation, coagulation, fibrinolysis, and endothelial dysfunction in the early stages of respiratory failure diagnosis (within 6 h post-ED presentation).

To summarise, AHRF patients exhibited higher levels of sEPCR and soluble PLG compared to the control group; however, these levels could not prognosticate the subsequent development of ARDS, using both the Berlin definition and the newly proposed global definition, nor worse outcomes.

## 5. Conclusions

Coagulation and fibrinolysis are known to be markedly abnormal in ARDS and independently associated with adverse clinical outcomes. Herein, we demonstrated that abnormalities in coagulation and fibrinolysis seem to be implicated in AHRF, highlighting the need for further research to fully understand the contribution of sEPCR and PLG. AHRF symptoms progress rapidly; hence, detecting early signs of dysregulated pathways could provide better clinical management, risk stratification, and potential therapeutic targets.

## Figures and Tables

**Table 1:** Demographics, airway management, biochemical data, and outcomes of the study population.

Characteristics	
Number of patients, *n*	130
Age (years), (median, IQR)	67 (59–73)
Sex, *n* (%)	
Male	63 (48.5)
Female	67 (51.5)
Comorbidities, *n* (%)	102 (78.5)
Hypertension	56
Chronic liver disease	33
Cancer	30
Diabetes	28
Heart condition	22
Hyperlipidaemia	20
Chronic kidney disease	9
Asthma/COPD exacerbation	3
Current medication, *n* (%)	51 (39.2)
Statins	31
Aspirin	17
Chemotherapy	8
Steroids	8
Amiodarone	1
PD-1/PDL-1 inhibitors	1
qSOFA, *n* (%)	
0	74 (56.9)
1	40 (30.8)
2	14 (10.8)
3	2 (1.5)
Vital signs	
Temperature (°C), (median, IQR)	36.7 (36.5–37.6)
Mean arterial pressure (mmHg), (median, IQR)	88 (80–96)
Airway management	
Other mean of oxygenation	85
Nasal canula	24
Venturi	19
Intubation	1
HFNC	1
Arterial blood gases	
SpO_2_ (%), (mean ± SD)	91 ± 5
PaO_2_ (mmHg), (median, IQR)	60 (53–68)
PaCO_2_ (mmHg), (median, IQR)	34.3 (30.2–39.2)
HCO_3_ (mEq/L), (mean ± SD)	24.9 ± 3.9
pH, (median, IQR)	7.45 (7.40–7.49)
SaO_2_ (%), (mean ± SD)	90.7 ± 5.8
Lactate (mmol/L), (median, IQR)	1.1 (0.8–1.7)
Predisposing risk factors, *n* (%)	129 (99.2)
SARS-CoV-2 infection	73
Pulmonary infection	36
Other	19
Non-pulmonary infection	4
Asthma/COPD exacerbation	3
Trauma/burns	1
Drugs	1
ED disposition	
General ward	103 (79.2)
ICU	27 (20.8)
Laboratory date	
Haematocrit, (mean ± SD)	38.2 ± 6.6
Haemoglobin, (mean ± SD)	12.6 ± 2.6
White blood cell count (per μL), (median, IQR)	9360 (6080–13,550)
Neutrophils (%), (median, IQR)	78.9 (69.6–86.4)
Lymphocytes (%), (median, IQR)	12.4 (7.6–20.5)
Platelets (per μL), (mean ± SD)	239,200 ± 100,800
PT (s), (median, IQR)	12.6 (12.0–13.6)
APTT (s), (median, IQR)	32.3 (29.1–35.6)
INR, (median, IQR)	1.08 (1.02–1.15)
Glucose (mg/dL), (median, IQR)	112 (98–143)
Urea (mg/dL), (median, IQR)	37 (27–53)
Creatinine (mg/dL), (median, IQR)	0.9 (0.7–1.2)
AST (IU/L), (median, IQR)	29.5 (19.0–49.8)
ALT (IU/L), (median, IQR)	22 (13–38)
ALP (U/L), (median, IQR)	78 (61–105)
γ-GT (IU/L), (median, IQR)	29 (17–58)
LDH (U/L), (median, IQR)	326 (234–504)
CK (U/L), (median, IQR)	88 (49–172)
CKMB (IU/L), (median, IQR)	28 (19–50)
CRP (mg/dL), (median, IQR)	8.8 (3.7–16.9)
Troponin (ng/mL), (median, IQR)	14 (8–30)
Outcomes	
Oxygen days (days), (median, IQR)	9 (4–20)
Mechanical ventilation, *n* (%)	29 (22.3)
Day of intubation, (median, IQR)	2 (1–8)
Duration of mechanical ventilation (days), (median, IQR)	11 (5–20)
HFNC, *n* (%)	34 (26.2)
Duration of HFNC (days), (median, IQR)	5 (2–10)
LoS in the ICU (days), (median, IQR)	16 (9–23)
ARDS, *n* (%)	29 (22.3)
LoS in the hospital (days), (median, IQR)	11 (8–19)
28-day hospital mortality, *n* (%)	20 (15.4)

Data are presented as the number of patients (*n*), percentages of total related variable (%), and mean ± SD for normally distributed variables or median (IQR) for skewed data. Measurements were performed within 6 h from ED presentation. ARDS was defined according to the Berlin definition. Definition of abbreviations: γ-GT = γ-Glutamyl transpeptidase; ALP = alkaline phosphatase; ALT = alanine transaminase; APTT = activated partial thromboplastin time; AST = aspartate transaminase; CK = creatine kinase; CKMB = creatinine kinase myocardial band; COPD = chronic obstructive pulmonary disease; CRP = C-reactive protein; ED = emergency department; HFNC = high-flow nasal canula; ICU = intensive care unit; INR = international normalised ratio; LDH = lactate dehydrogenase; LoS = length of stay; PaCO_2_ = partial pressure of carbon dioxide; PaO_2_ = partial pressure of oxygen; PD-1 = programmed death-1; PDL-1 = programmed death-ligand 1; PT = prothrombin time, qSOFA = quick sequential organ failure assessment; SaO_2_ = arterial oxygen saturation; SpO_2_ = oxygen saturation; SARS-CoV-2 = severe acute respiratory syndrome coronavirus 2.

## Data Availability

Data are available from the corresponding author upon reasonable request.
